# Association between the ROX index and mortality in patients with acute hypoxemic respiratory failure: a retrospective cohort study

**DOI:** 10.1186/s12931-024-02771-9

**Published:** 2024-03-29

**Authors:** Kai Liu, Xin-Yi Ma, Hua Xiao, Wan-Jie Gu, Jun Lyu, Hai-Yan Yin

**Affiliations:** 1 Department of Intensive Care Unit, The First Affiliated Hospital of Jinan University, 613 Huangpu Avenue West, Guangzhou, 510630 China; 2 The First Clinical Medical College, Southern Medical University, Guangzhou, China; 3 Department of Nephrology, Shenzhen People’s Hospital (The Second Clinical Medical College of Jinan University, The First Affiliated Hospital of Southern University of Science and Technology), Shenzhen, China; 4 Department of Clinical Research, The First Affiliated Hospital of Jinan University, Guangzhou, Guangdong Province China

**Keywords:** ROX index, Acute hypoxemic respiratory failure, Mortality, MIMIC-IV

## Abstract

**Background:**

Although ROX index is frequently used to assess the efficacy of high-flow nasal cannula treatment in acute hypoxemic respiratory failure (AHRF) patients, the relationship between the ROX index and the mortality remains unclear. Therefore, a retrospective cohort study was conducted to evaluate the ability of the ROX index to predict mortality risk in patients with AHRF.

**Method:**

Patients diagnosed with AHRF were extracted from the MIMIC-IV database and divided into four groups based on the ROX index quartiles. The primary outcome was 28-day mortality, while in-hospital mortality and follow-up mortality were secondary outcomes. To investigate the association between ROX index and mortality in AHRF patients, restricted cubic spline curve and COX proportional risk regression were utilized.

**Result:**

A non-linear association (L-shaped) has been observed between the ROX index and mortality rate. When the ROX index is below 8.28, there is a notable decline in the 28-day mortality risk of patients as the ROX index increases (HR per SD, 0.858 [95%CI 0.794–0.928] *P* < 0.001). When the ROX index is above 8.28, no significant association was found between the ROX index and 28-day mortality. In contrast to the Q1 group, the mortality rates in the Q2, Q3, and Q4 groups had a substantial reduction (Q1 vs. Q2: HR, 0.749 [0.590–0.950] *P* = 0.017; Q3: HR, 0.711 [0.558–0.906] *P* = 0.006; Q4: HR, 0.641 [0.495–0.830] *P* < 0.001).

**Conclusion:**

The ROX index serves as a valuable predictor of mortality risk in adult patients with AHRF, and that a lower ROX index is substantially associated with an increase in mortality.

## Introduction

Acute hypoxemic respiratory failure (AHRF) is one of the prominent causes of hospitalization in intensive care units (ICU) [1]. The findings of a multicenter observational study conducted in over 50 countries worldwide revealed that AHRF constituted around one-third of patients necessitating mechanical ventilation, and the death rate associated with AHRF frequently surpassed 40% [2], and the prevalence of COVID-19 underscores the gravity of this illness [3, 4]. The occurrence of AHRF imposes a substantial burden on both families and society. Therefore, the ability to accurately estimate the likelihood of mortality in advance holds significant importance in guiding appropriate medical interventions. The Respiratory Extracorporeal Membrane Oxygenation Survival Prediction (RESP) score has the potential to serve as a prognostic tool for assessing mortality risk in individuals diagnosed with acute respiratory failure [5]. However, its application is limited to patients who have undergone extracorporeal membrane oxygenation (ECMO) treatment for respiratory failure. And the prognostic value of the oxygenation index (PaO_2_/FiO_2_ ratio) is limited to children with AHRF, while its efficacy in adult patients is suboptimal [6, 7]. Hence, there is a pressing need for a prognostic tool that can accurately anticipate the outcome of adult individuals suffering from AHRF. Such a tool would assist clinical practitioners in identifying patients at a heightened risk of mortality, enabling them to implement more focused and proactive treatment strategies.

The ROX index is defined as the ratio between oxygen saturation and the percentage of inspired oxygen (SpO_2_/FiO_2_) and the respiratory rate (RR). In recent years, this index has gained significant popularity as a result of its convenient use in bedside detection [8]. The characteristics assessed by this index are non-intrusive and can be measured at any given moment, and it can even be measured by non-healthcare personnel. Currently, the ROX index is widely employed as a means of assessing the potential failure of High Flow Nasal Cannula (HFNC) therapy in patients with AHRF [9, 10], and as a convenient and real-time monitoring index, we have a curiosity to find out whether the ROX index can offer more information into patients with AHRF. Consequently, a retrospective cohort analysis was undertaken to validate the accuracy of the ROX index in prognosticating mortality.

## Method

### Study design

We employed a retrospective cohort approach to investigate the association between the ROX index and mortality among adult patients with AHRF in ICU. The data utilized in this study was obtained from the MIMIC-IV version 2.0 database. The present database encompasses the electronic health records of a substantial cohort of more than 50,000 patients who were admitted to the ICU at Beth Israel Deacon Medical Center (Boston, Massachusetts, USA) over the period spanning from 2008 to 2019, and it has received approval from the Beth Israel Deacon Medical Center and the Massachusetts Institute of Technology Institutional Review Board. The data has been de-identified and made publicly accessible, eliminating the requirement for specific informed permission from patients. The author (KL) gained dataset access through an examination (Record ID 12,102,940), extracted data using Structured Query Language (SQL), and performed statistical analysis using the R programming language software. The present investigation was conducted in adherence to the guidelines outlined in the Strengthening the Reporting of Observative Studies in Epidemiology (STROBE) statement.

**Fig. 1:**
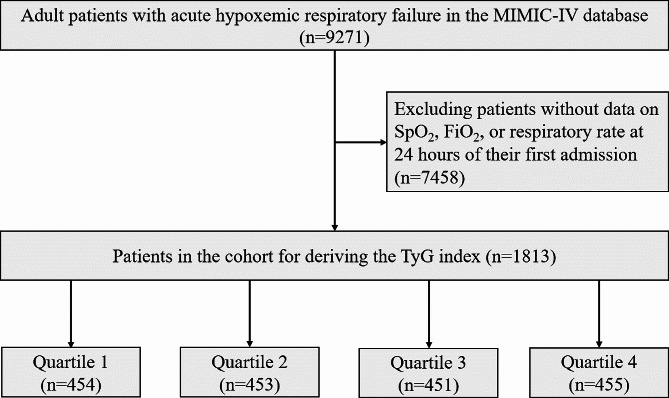
Flowchart of patient selection

A total of 9271 adult patients diagnosed with AHRF were included in our study. Patients with missing data, such as SpO_2_, FiO_2_, or RR on the first day of admission, were excluded from the analysis. For patients who entered the ICU multiple times, only data from the initial admission were utilized. The study cohort ultimately consisted of 1813 patients and was categorized into four groups according to the quartiles of the ROX index upon initial admission. The patient screening process diagram is shown in Fig. 1.

### Clinical outcomes

This study’s primary outcome was 28-day all-cause mortality, with secondary outcomes including 3-month mortality, 6-month mortality, 1-year mortality, in-ICU mortality, and in-hospital mortality.

### Variable extraction

We gathered patient information during hospitalization and follow-up, including age, gender, race, BMI, SOFA score, SIRS score, SAPS II score, Charles comorbidities index, average vital signs (heart rate, RR, mean blood pressure, oxygen saturation, oxygenation index) recorded within the initial 24-hour period, and comorbidities (myocardial infarct, cerebrovascular disease, congestive heart failure, peripheral vascular disease, chronic pulmonary disease, diabetes, severe liver disease, renal disease, malignant cancer), first laboratory tests results (WBC, platelet, albumin, sodium, chloride, potassium, calcium, glucose, AST, ALT, BUN, creatinine, INR, PT, PTT), and we calculate the ROX index using the average values of the SpO_2_, FiO_2_, and RR measurements taken within 24 hours of patient admission. To mitigate the potential bias resulting from sample removal, we employed multiple imputation techniques using the “mice” package in the R program for variables with missing data below 20%, and covariates with missing data over 20% were excluded from the model.

### Statistical analysis

Continuous variables were denoted by their median value together with the interquartile range (IQR), to assess the differences between two groups, the Mann-Whitney U test was employed. Categorical variables were quantified as numerical percentages and subsequently analyzed by statistical tests such as the Chi-square test or Fisher’s exact test for comparison. Employing the RCS curve, the nonlinear relationship between ROX index and mortality was evaluated. Consequently, a piecewise linear model was developed using the curve results to quantify the relationship [11, 12]. Based on whether or not their ROX index exceeded the median value, the patients were classified into two categories. The HR values were then calculated using Cox regression. Furthermore, we performed subgroup analysis on patients with ROX index below the median to evaluate the consistency of the influence of the ROX index on the primary outcome. The primordial population was divided into subgroups based on age (> 65 years and ≤ 65 years), gender (male and female), and body mass index (≥ 30 kg/m^2^ and < 30 kg/m^2^).

The patients were categorized into four groups according to the quartiles of the ROX index. The incidence of outcome events was assessed using Kaplan Meier survival analysis, and the differences between the groups were evaluated using the log-rank test. We employed the Cox proportional hazards model to calculate the hazard ratio (HR) and 95% confidence interval (CI) for the association between the ROX index and the outcome, the Schoenfeld residual test was utilized to assess the validity of the proportional risk hypothesis. Variables that exhibited a *P*-value greater than 0.05 were deemed to conform to the hypothesis.

The baseline variable was considered as a potential predictor variable in the Cox proportional hazards model. In order to address the potential issue of overfitting, the variance inflation factor (VIF) was employed as a measure to assess the presence of multicollinearity among variables. And variables with a VIF value of 5 or above were subsequently eliminated from the analysis. Based on clinical expertise and previous studies, as well as the outcomes of univariate Cox regression analysis, the following variables were incorporated into the multivariate Cox proportional risk regression model: age, gender, and race, Charlson comorbidity index, cerebrovascular disease, malignant cancer, severe liver disease, white blood cells, potassium, sodium, calcium, glucose, albumin, aspartate aminotransferase, alanine aminotransferase, blood urea nitrogen, creatinine, international normalized ratio, prothrombin time, partial thromboplastin time, high-flow nasal cannula, and invasive vent.

The statistical studies were conducted using R version 4.1.0, a two-sided *P*-value less than 0.05 was deemed to be statistically significant in this study.

## Result

1813 patients were included in the study, with an average age of 67 (55, 77) years and 1068 males (58.9%). All patients had an average ROX index of 8.3 (5.9, 11.3). During the period of hospitalization, a total of 276 patients (15.2%) died within the ICU, while 481 patients (26.5%) died in the hospital. During the follow-up period, 513 patients (28.3%) died within 28 days after admission, 640 patients (35.3%) died within 3 months, 705 patients (38.9%) died within 6 months, and 775 patients (42.7%) died within 1 year. (Table 1)

**Table 1:** Baseline characteristics of patients with acute hypoxemic respiratory failure according to the ROX index

Categories	Overall (*N* = 1813)	Q1 (*N* = 454)	Q2 (*N* = 453)	Q3 (*N* = 451)	Q4 (*N* = 455)	*P*-value
ROX index	8.3 (5. 9, 11.3)	4.5 (3.7, 5.3)	7 (6.4, 7.7)	9.6 (8.9, 10.3)	13.6 (12.4, 15.6)	**< 0.001**
Age (year)	67 (55, 77)	64 (53, 74)	69 (57, 78)	67 (56, 78)	68 (56, 80)	**< 0.001**
**Gender**						
Male	1068 (58.9%)	266 (58.6%)	269 (59.4%)	273 (60.5%)	260 (57.1%)	0.768
Female	745 (41.1%)	188 (41.4%)	184 (40.6%)	178 (39.5%)	195 (42.9%)	
**Race**						
White	1047 (57.7%)	253 (55.7%)	266 (58.7%)	264 (58.5%)	264 (58%)	0.210
Black	166 (9.2%)	32 (7%)	47 (10.4%)	39 (8.6%)	48 (10.5%)	
Asian	56 (3.1%)	10 (2.2%)	11 (2.4%)	21 (4.7%)	14 (3.1%)	
Other	129 (7.1%)	35 (7.7%)	31 (6.8%)	33 (7.3%)	30 (6.6%)	
Unknown	415 (22.9%)	124 (27.3%)	98 (21.6%)	94 (20.8%)	99 (21.8%)	
BMI (kg/m^2^)	28 (23, 32)	28 (24, 33)	29 (24, 33)	28 (23, 32)	26 (22, 31)	**< 0.001**
SOFA	9 (6, 12)	11 (8, 14)	9 (6, 12)	8 (6, 11)	7 (4, 9)	**< 0.001**
SIRS	3 (2, 4)	3 (3, 4)	3 (2, 4)	3 (2, 3.5)	3 (2, 3)	**< 0.001**
SAPS II	41 (33, 52)	48 (38, 61)	43 (34, 53)	40 (31.5, 50)	37 (29, 46)	**< 0.001**
Charlson comorbidity index	6 (4, 8)	6 (4, 8)	6 (4, 8)	7 (4, 9)	6 (4, 8)	**0.004**
**Vital signs**						
Heart rate (beats/min)	84.6 (75.3, 98.2)	89.5 (79.3, 104.6)	84.3 (74.4, 97.7)	84.1 (75.2, 98.1)	80.8 (72.0, 92.0)	**< 0.001**
Respiratory rate (breaths/min)	19.7 (17.3, 22.9)	23.1 (20.3, 26.1)	20.4 (18.0, 23.3)	19.3 (17.5, 21.2)	17.2 (15.6, 19.0)	**< 0.001**
MBP (mmHg)	75.6 (70.5, 81.7)	74.0 (68.8, 80.0)	74.7 (70.2, 80.9)	75.9 (70.8, 81.6)	77.5 (71.7, 84.1)	**< 0.001**
SpO_2_ (%)	97.6 (96.0, 98.8)	96.2 (94.5, 97.8)	97.4 (96.1, 98.5)	97.9 (96.6, 99.0)	98.4 (97.3, 99.3)	**< 0.001**
PaO_2_/FiO_2_	114 (74, 198)	78.8 (55, 118.7)	115 (77.5, 184)	122.5 (82.5, 206)	164 (102.7, 291.3)	**< 0.001**
**Comorbidities**						
Myocardial infarct	454 (25%)	115 (25.3%)	115 (25.4%)	129 (28.6%)	95 (20.9%)	0.063
Cerebrovascular disease	325 (17.9%)	67 (14.8%)	79 (17.4%)	80 (17.7%)	99 (21.8%)	0.052
Congestive heart failure	689 (38%)	157 (34.6%)	197 (43.5%)	176 (39%)	159 (34.9%)	**0.018**
Peripheral vascular disease	195 (10.8%)	56 (12.3%)	54 (11.9%)	41 (9.1%)	44 (9.7%)	0.298
Chronic pulmonary disease	485 (26.8%)	125 (27.5%)	127 (28%)	118 (26.2%)	115 (25.3%)	0.775
Diabetes	591 (32.6%)	131 (28.9%)	165 (36.4%)	151 (33.5%)	144 (31.6%)	0.100
Severe liver disease	164 (9%)	52 (11.5%)	35 (7.7%)	39 (8.6%)	38 (8.4%)	0.211
Renal disease	479 (26.4%)	108 (23.8%)	123 (27.2%)	132 (29.3%)	116 (25.5%)	0.281
Malignant cancer	1549 (85.4%)	67 (14.8%)	60 (13.2%)	79 (17.5%)	58 (12.7%)	0.171
**Laboratory tests**						
WBC (K/uL)	15 (10.7, 20.8)	17 (11.3, 23.8)	16 (11.8, 22.2)	14.6 (11, 20.4)	13.2 (9.55, 17.15)	**< 0.001**
Platelet (K/uL)	151 (103, 209)	146 (88, 198.75)	153 (101, 212)	153 (109.5, 214)	156 (109, 210)	0.051
Albumin (g/dL), mean ± sd	3.2 ± 0.7	3.1 (2.6, 3.6)	3.2 (2.7, 3.6)	3.2 (2.6, 3.7)	3.3 (2.75, 3.7)	**0.014**
Sodium (mEq/L)	137 (134, 140)	137 (133, 139)	138 (134, 140)	138 (134, 140)	138 (135, 140)	**0.005**
Chloride (mEq/L)	105 (101, 108)	105 (101, 108)	104 (100, 107)	105 (102, 108)	105 (101, 108)	0.208
Potassium (mEq/L)	4.5 (3.9, 5.3)	4.6 (4, 5.4)	4.6 (4, 5.4)	4.5 (3.8, 5.4)	4.4 (3.8, 5.1)	**0.033**
Calcium (mEq/L)	1.06 (0.99, 1.12)	1.04 (0.96, 1.1)	1.06 (0.98, 1.12)	1.06 (1, 1.12)	1.07 (1.01, 1.12)	**< 0.001**
Glucose (mg/dL)	136.0 (114.2, 170.3)	141.4 (116.8, 178.9)	139 (118, 172.6)	135.6 (114.3, 170)	130 (110.1, 158.8)	**< 0.001**
AST (IU/L)	54 (28, 152)	71 (35, 251.5)	59 (31, 150)	48 (27, 139.5)	45 (25, 105)	**< 0.001**
ALT (IU/L)	31 (18, 85)	38 (20, 116.5)	37 (19, 90)	31 (16, 92.5)	27 (16, 55.5)	**< 0.001**
BUN (mg/dL)	25 (17, 41)	28 (19, 48)	26 (18, 42)	24 (17, 37)	22 (14, 33)	**< 0.001**
Creatinine (mg/dL)	1.3 (0.9, 2.1)	1.7 (1, 2.5)	1.3 (0.9, 2.1)	1.3 (0.9, 2)	1 (0.8, 1.6)	**< 0.001**
INR	1.4 (1.2, 1.7)	1.5 (1.3, 1.9)	1.4 (1.2, 1.7)	1.3 (1.2, 1.6)	1.2 (1.1, 1.5)	**< 0.001**
PT (s)	14.8 (12.8, 18.6)	16 (13.6, 21.7)	15 (13.1, 18.4)	14.7 (12.9, 17.7)	13.6 (12.2, 16.2)	**< 0.001**
PTT (s)	32.8 (28.3, 46.9)	36.3 (29.7, 55.7)	33.3 (28.7, 47.1)	32.4 (28.2, 44.3)	31 (27.4, 39.9)	**< 0.001**
**Ventilation status**						
HFNC	195 (10.8%)	78 (17.2%)	47 (10.4%)	35 (7.8%)	35 (7.7%)	**< 0.001**
Invasive Vent	1579 (87.1%)	414 (91.2%)	414 (91.4%)	390 (86.5%)	361 (79.3%)	**< 0.001**
Non-Invasive Vent	116 (6.3%)	30 (6.6%)	40 (8.8%)	24 (5.3%)	22 (4.8%)	0.065
Supplemental Oxygen	1326 (73.1%)	311 (68.5%)	344 (75.9%)	331 (73.4%)	340 (74.7%)	0.061
Tracheostomy	115 (6.3%)	32 (7%)	30 (6.6%)	24 (5.3%)	29 (6.4%)	0.745
**Outcomes**						
28-day mortality	513 (28.3%)	157 (34.6%)	127 (28%)	124 (27.5%)	105 (23.1%)	**0.002**
3-month mortality	640 (35.3%)	195 (43%)	153 (33.8%)	156 (34.6%)	136 (29.9%)	**< 0.001**
6-month mortality	705 (38.9%)	205 (45.2%)	174 (38.4%)	175 (38.8%)	151 (33.2%)	**0.003**
1-year mortality	775 (42.7%)	217 (47.8%)	198 (43.7%)	185 (41%)	175 (38.5%)	**0.031**
In-ICU mortality	276 (15.2%)	113 (24.9%)	69 (15.2%)	58 (12.9%)	36 (7.9%)	**< 0.001**
In-hospital mortality	481 (26.5%)	160 (35.2%)	121 (26.7%)	109 (24.2%)	91 (20%)	**< 0.001**
Length of hospital stay (d)	12.7 (6.7, 21.4)	13.7 (7.7, 22.8)	12.7 (7.1, 21.4)	11.7 (6.7, 21.5)	11.5 (5.8, 20.0)	0.104
Length of ICU stay (d)	4.9 (2.4, 9.9)	6.14 (2.8, 12.2)	5.2 (3.0, 9.9)	4.8 (2.4, 9.2)	3.7 (2.0, 8.2)	**< 0.001**

Data were presented as the median with interquartile range for continuous variables and number with frequency for categorical variables unless otherwise indicated

BMI, Body Mass Index; SOFA, Sequential Organ Failure Assessment; SIRS, Systemic inflammatory response syndrome; SAPS II, Simplified acute physiological score II; MBP, mean blood pressure; ALT, alanine aminotransferase; AST, aspartate aminotransferase; BUN, blood urea nitrogen; INR, International normalized ratio; PT, prothrombin time; PTT, partial thromboplastin time

### Baseline characteristics

Patients were categorized into four groups (Q1: ROX ≤ 5.89; Q2: 5.89 < ROX ≤ 8.28; Q3: 8.28 < ROX ≤ 11.24; Q4: ROX > 11.24) based on the quartiles of the ROX index, and the baseline characteristics of each group are shown in Table 1. Compared to the group with lower ROX index, patients with higher ROX index had lower admission severity scores (SOFA, SIRS, SAPS II), lower heart and respiratory rates, higher MBP, SpO_2_, PaO_2_/FiO_2_, and were more likely to be receiving HFNC and invasive ventilation. As the ROX index increases, there is a gradual decrease in various mortality rates, including the 28-day mortality rate (34.6% vs. 28% vs. 27.5% vs. 23.1%), 1-year mortality rate (47.8% vs. 43.7% vs. 41% vs. 38.5%), in-ICU mortality rate (24.9% vs. 15.2% vs. 12.9% vs. 7.9%), and in-hospital mortality rate (35.2% vs. 26.7% vs. 24.2% vs. 20%). Additionally, there is a gradual decrease in the length of ICU stay (6.14 days vs. 5.2 days vs. 4.8 days vs. 3.7 days, *P* < 0.001) (Table 1).

### Association between ROX index and all-cause mortality rate

**Fig. 2:**
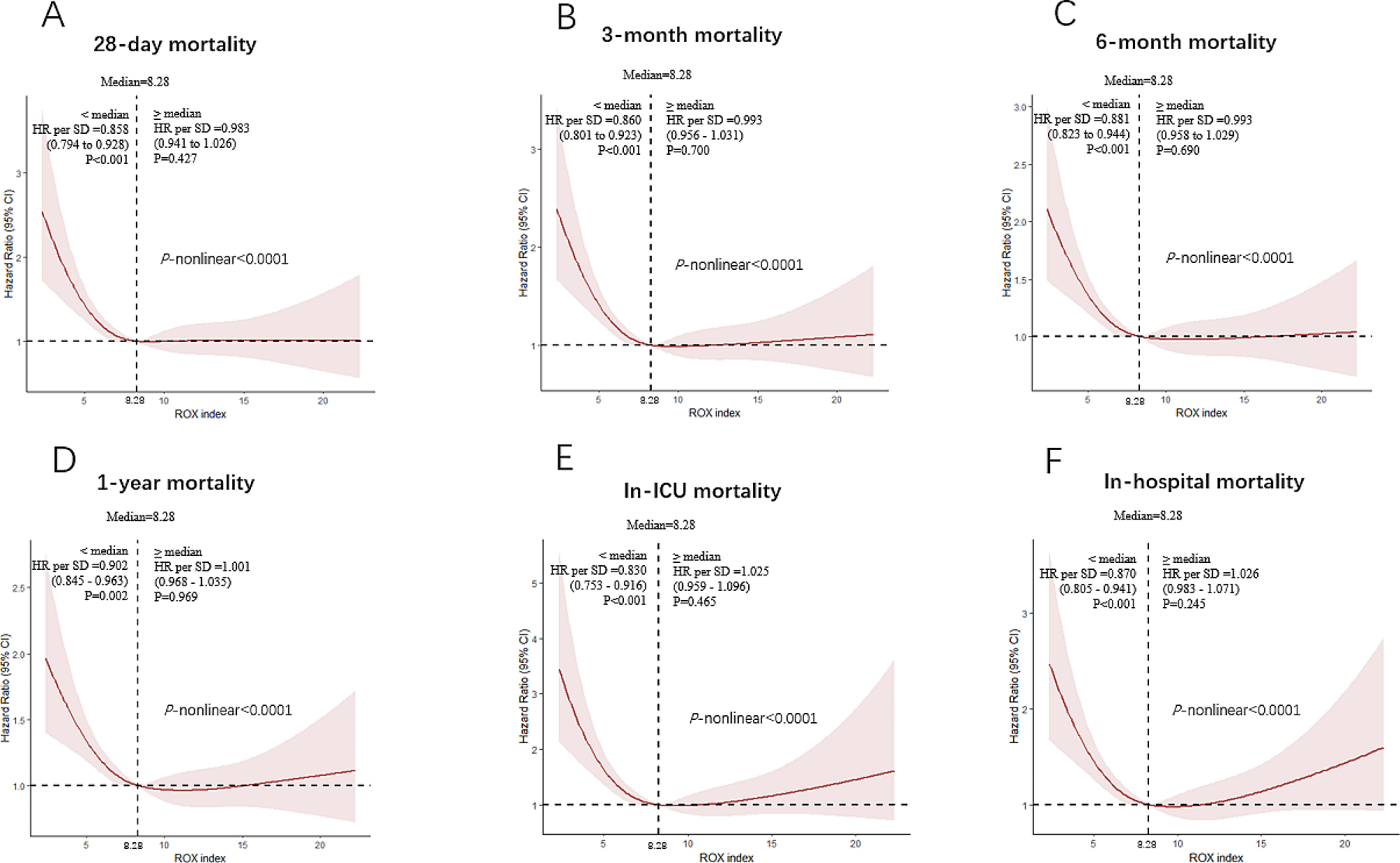
Restricted cubic spline (RCS) curve of the ROX index and HR in patients with acute hypoxemic respiratory failure. (**A**) RCS curve for 28-day mortality. (**B**) RCS curve for 3-month mortality. (**C**) RCS curve for 6-month mortality. (**D**) RCS curve for 1-year mortality. (**E**) RCS curve for in-ICU mortality. (**F**) RCS curve for in-hospital mortality

Restricted cubic spline curve was employed in order to flexibly visualize and analyze the association between the ROX index and all-cause mortality in individuals diagnosed with AHRF. Whether the patient is hospitalized or in the follow-up phase, there is an L-shaped relationship between the ROX index and mortality (Fig. 2). For quantifying the correlation, we fitted a simple piecewise linear model. When the ROX index is below 8.28, there is a notable decline in the 28-day mortality risk of patients as the ROX index increases (HR per SD, 0.858 [95%CI 0.794–0.928] *P* < 0.001). When the ROX index is above 8.28, the risk curve for all-cause mortality remains stable, and there was no significant link found between the patient’s ROX index and the 28-day mortality rate (HR per SD, 0.983 [95%CI 0.941–1.026] *P* = 0.427). Similar results were observed in Cox proportional risk analysis of the ROX index and 3-month mortality, 6-month mortality, 1-year mortality, in-ICU mortality and in-hospital mortality.

**Fig. 3:**
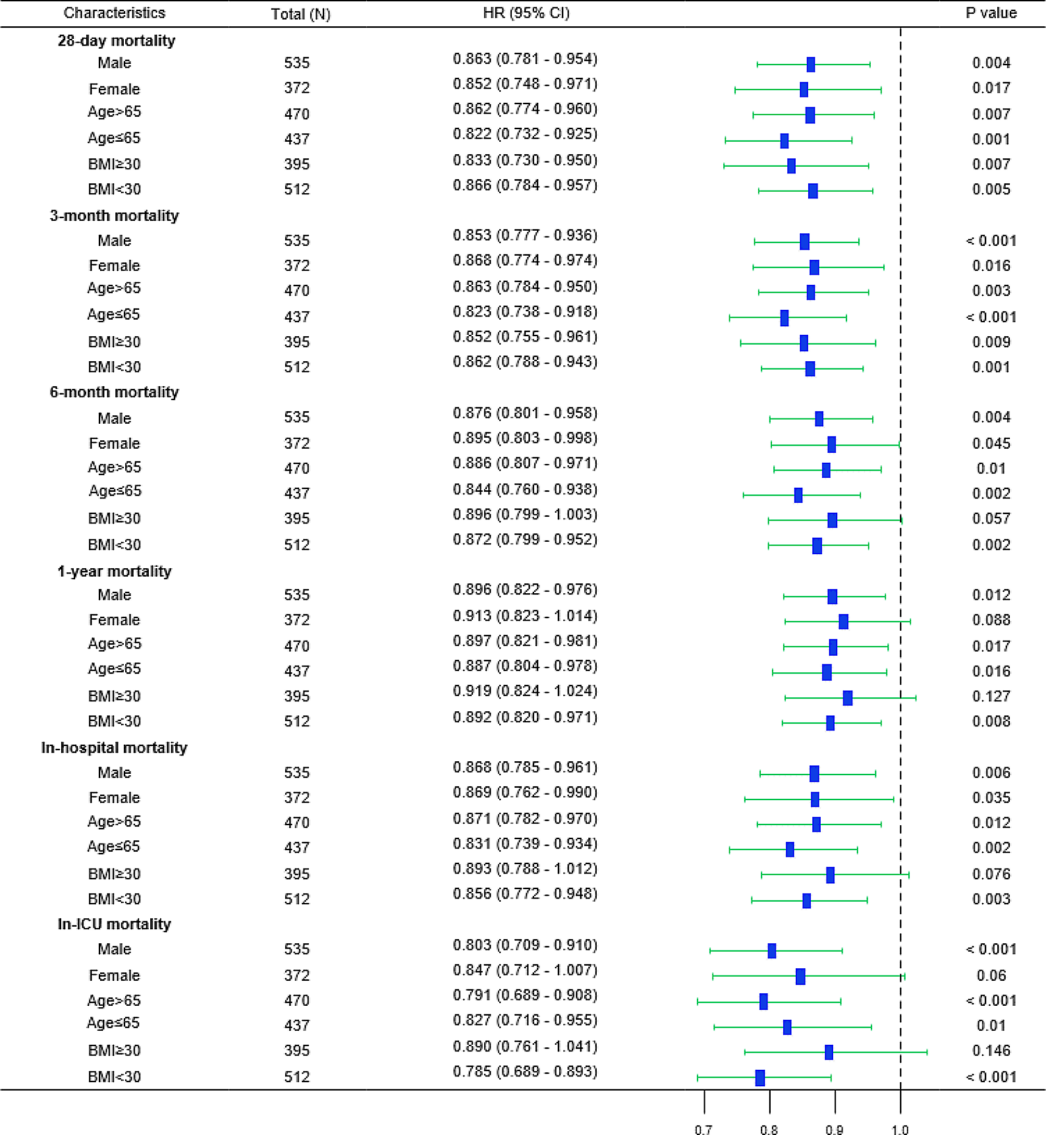
Forest plots of all-cause mortality in subgroups

In addition, patients with a ROX index below 8.28 were categorized into various subgroups according to characteristics such as age, gender, and BMI, and we noticed that the ROX index was substantially correlated with 28-day mortality in subgroups including male (HR [95% CI] 0.863 [0.781–0.954]), female (HR [95% CI] 0.852 [0.748–0.971]), age > 65 years (HR [95% CI] 0.862 [0.774–0.960]), age ≤ 65 years (HR [95% CI] 0.822 [0.732–0.925]), BMI ≥ 30 kg/m^2^ (HR [95% CI] 0.833 [0.730–0.950]), and BMI < 30 kg/m^2^ (HR [95% CI] 0.866 [0.784–0.957]). Furthermore, a substantial correlation between the ROX index and all cause death was observed in nearly all subgroups of secondary outcome measures (Fig. 3).

### Disparity in mortality between various groups

**Fig. 4:**
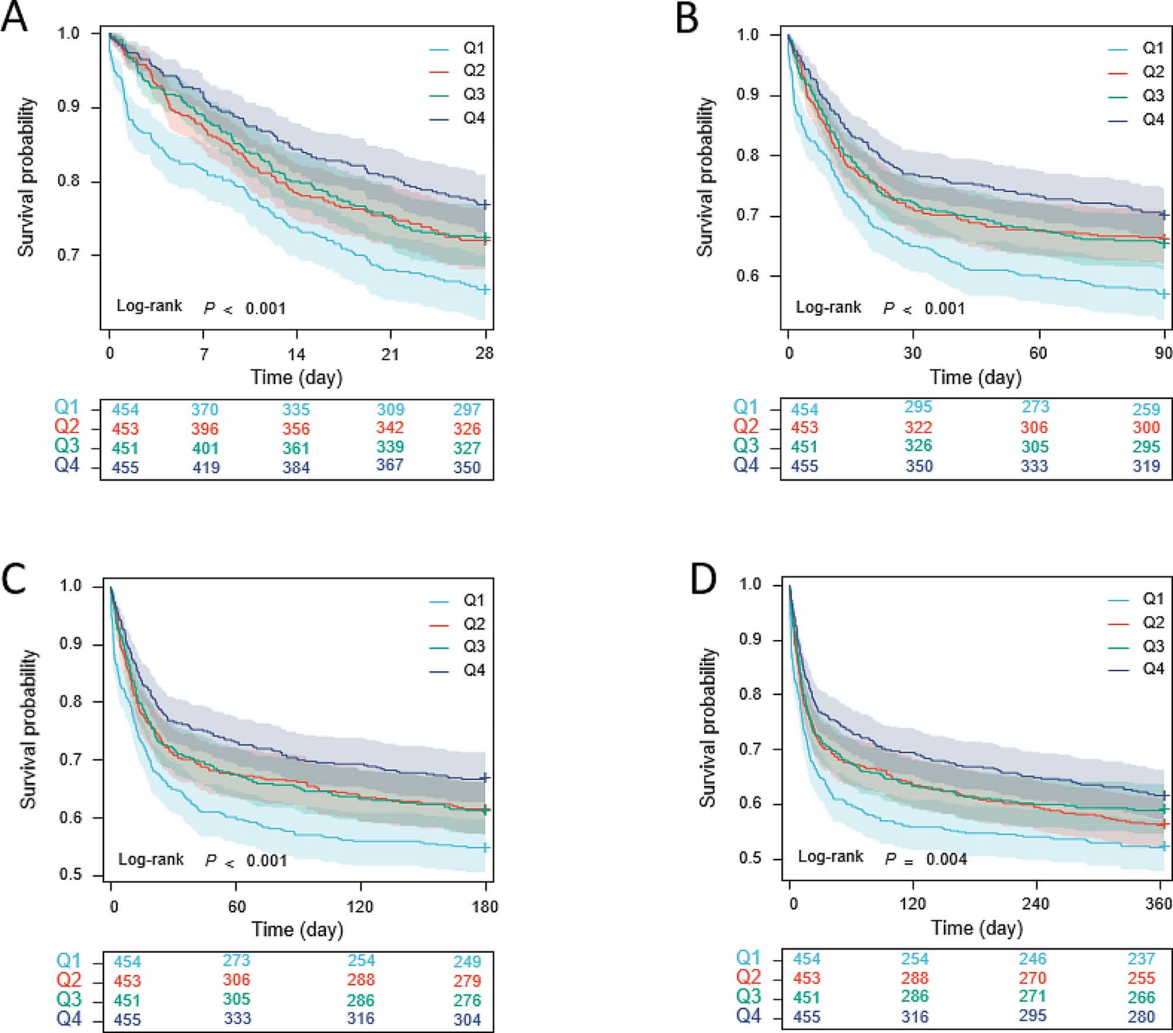
Kaplan–Meier survival analysis curves for all-cause mortality. ROX index: Q1 (ROX ≤ 5.89), Q2 (5.89 < ROX ≤ 8.28), Q3 (8.28 < ROX ≤ 11.24), Q4 (ROX > 11.24). Kaplan–Meier curves showing cumulative probability of all-cause mortality according to groups at 28 days (**A**), 3 months (**B**), 6 months (**C**) and 1 year (**D**)

Patients were divided into four groups according to the quartiles of the ROX index, the Kaplan-Meier survival analysis curves for each group are shown in Fig. 4. Within 28 days of admission, the all-cause mortality rates of patients in the Q2, Q3, and Q4 groups were substantially lower than those of patients in the Q1 group (34.6% vs. 28.0% vs. 27.5% vs. 23.1%, log rank *P* < 0.001). Besides, there were statistically significant disparities in mortality rates across the various groups during the 3-month, 6-month, and 1-year observation periods (all log rank *P* < 0.01) (Fig. 4).

And the Cox proportional hazards analysis revealed statistically significant variations in the 28-day death rates among various groups, as shown in both the unadjusted (Q1 vs. Q2: HR, 0.754 [0.597–0.953] *P* = 0.018; Q3: HR, 0.735 [0.581–0.930] *P* = 0.010; Q4: HR, 0.595 [0.465–0.762] *P* < 0.001) and adjusted models (Q1 vs. Q2: HR, 0.749 [0.590–0.950] *P* = 0.017; Q3: HR, 0.711 [0.558–0.906] *P* = 0.006; Q4: HR, 0.641 [0.495–0.830] *P* < 0.001). Similar results were also observed during follow-up periods of 3 months, 6 months, and 1 year (Table 2).

**Table 2:** Cox proportional hazard ratios (HR) for all-cause mortality

Categories	Deaths (%)	Unadjusted model		Adjusted model	
		HR (95% CI)	*P*-value	HR (95% CI)	*P*-value
**28-day mortality**					
Q1 (*N* = 454)	157 (34.6%)	reference		reference	
Q2 (*N* = 453)	127 (28%)	0.754 (0.597–0.953)	0.018	0.749 (0.590–0.950)	0.017
Q3 (*N* = 451)	124 (27.5%)	0.735 (0.581–0.930)	0.010	0.711 (0.558–0.906)	0.006
Q4 (*N* = 455)	105 (23.1%)	0.595 (0.465–0.762)	< 0.001	0.641 (0.495–0.830)	< 0.001
**3-month mortality**					
Q1 (*N* = 454)	195 (43%)	reference		reference	
Q2 (*N* = 453)	153 (33.8%)	0.726 (0.587–0.897)	0.003	0.705 (0.568–0.874)	0.001
Q3 (*N* = 451)	156 (34.6%)	0.739 (0.599–0.912)	0.005	0.690 (0.555–0.857)	< 0.001
Q4 (*N* = 455)	136 (29.9%)	0.611 (0.491–0.760)	< 0.001	0.640 (0.509–0.804)	< 0.001
**6-month mortality**					
Q1 (*N* = 454)	205 (45.2%)	reference		reference	
Q2 (*N* = 453)	174 (38.4%)	0.782 (0.639–0.957)	0.017	0.753 (0.613–0.925)	0.007
Q3 (*N* = 451)	175 (38.8%)	0.786 (0.642–0.962)	0.019	0.723 (0.587–0.890)	0.002
Q4 (*N* = 455)	151 (33.2%)	0.641 (0.519–0.791)	< 0.001	0.662 (0.531–0.824)	< 0.001
**1-year mortality**					
Q1 (*N* = 454)	217 (47.8%)	reference		reference	
Q2 (*N* = 453)	198 (43.7%)	0.840 (0.693–1.018)	0.076	0.801 (0.658–0.974)	0.026
Q3 (*N* = 451)	185 (41%)	0.783 (0.644–0.953)	0.015	0.710 (0.580–0.869)	< 0.001
Q4 (*N* = 455)	175 (38.5%)	0.699 (0.573–0.853)	< 0.001	0.708 (0.575–0.872)	0.001

Q1: (ROX ≤ 5.89), Q2: (5.89 < ROX ≤ 8.28), Q3: (8.28 < ROX ≤ 11.24), Q4: (ROX > 11.24)

HR: hazard ratio; CI: confidence interval;

Adjusted model: adjusted for age, gender, race, Charlson comorbidity index, cerebrovascular disease, malignant cancer, severe liver disease, white blood cell, potassium, sodium, calcium, glucose, albumin, aspartate aminotransferase, alanine aminotransferase, blood urea nitrogen, creatinine, international normalized ratio, prothrombin time, partial thromboplastin time, high flow nasal cannula, invasive vent

## Discussion

This study reveals a notable association between a decline in the ROX index and an escalation in mortality among patients with AHRF, the ROX index demonstrates its utility as an effective instrument for evaluating the mortality risk in individuals with AHRF.

The PaO_2_/FiO_2_ ratio is frequently employed in medicine to assess the oxygenation efficiency of individuals experiencing acute respiratory failure [13]. Interestingly, previous research has demonstrated a correlation between the SpO_2_/FiO_2_ ratio and the PaO_2_/FiO_2_ ratio [14]. The diagnostic precision of the SpO_2_/FiO_2_ ratio in individuals with acute respiratory distress syndrome (ARDS) has a comparable level of accuracy to that of the PaO_2_/FiO_2_ ratio [15]. AHRF is characterized by various signs, including structural damage to the lungs, oxygenation disorders, alterations in respiratory mechanics, and an elevation in the fraction of alveolar dead space, and it is generally believed that a direct correlation exists between the aggravation of hypoxemia and a resulting increase in mortality [1, 16, 17], several studies have also indicated that respiratory dysfunction may be correlated with unfavorable outcomes [18–20]. The ROX index integrates the oxygenation status (measured by SpO_2_/FiO_2_) and respiratory distress (measured by RR) of patients, whereas critically ill patients typically have both a lower SpO_2_/FiO_2_ and a higher RR [9]. Consequently, the ROX index may be a reliable predictor for patients with critical illness.

Previous studies have demonstrated that the PaO_2_/FiO_2_ ratio holds predictive value in assessing the prognosis of pediatric patients experiencing AHRF. However, it should be noted that in adult patients, the efficacy of the oxygenation index as a prognostic indicator is suboptimal, a prospective multicenter study showed that a low PaO_2_/FiO_2_ ratio was associated with 60-day mortality only in non-ARDS patients with hypoxemia [21]. In addition, the acquisition of PaO_2_ measurements from patients necessitates the extraction of arterial blood, a procedure that carries the risk of anemia, hemorrhage, vascular injury, and complications associated with surgical interventions [22]. The measurement of SpO_2_ can be achieved non-invasively by the utilization of a pulse oximeter, it allows for continuous monitoring, and enabling the early detection of mortality risk in patients experiencing hypoxic respiratory failure, for decades, continuous pulse oximetry has been a component of standard monitoring in intensive care units. Consequently, the utilization of ROX for monitoring hypoxemia in critically ill patients offers more benefits compared to arterial blood gas monitoring.

Prior research has frequently employed the ROX index at a particular time as a predictive indicator, however, it should be noted that the ROX index is susceptible to the influence of clinical variables and exhibits frequent fluctuations [23]. To enhance the precision of assessing hypoxia levels in patients, the ROX index was computed by taking the average of the parameters (SpO_2_, FiO_2_, RR) measured within a 24-hour period following admission, these measures may lead to a reduction in mistakes and enhance the predictive accuracy of the ROX index in assessing patient prognosis and mortality risk. Furthermore, our research demonstrates that the predictive efficacy of the ROX index diminishes upon surpassing the critical threshold, a reasonable explanation is that the death rate of patients exhibits a notable escalation just when the degree of hypoxia attains a relatively critical threshold.

The ROX index is a cost-effective bedside monitoring indicator that eliminates the need for expensive specialized equipment or complex laboratory testing. It only requires a standard monitor to track the patient’s SpO_2_, FiO_2_, and RR. This allows the ROX index to be widely used in resource-limited medical environments, reducing medical costs. At the same time, bedside testing reduces the medical risks associated with patient movement and assists the medical team in detecting changes in the patient’s condition in a timely manner, providing strong support for clinical decision making [8].

There are also some limitations to this investigation. Firstly, because it is a retrospective study, causal relationships cannot be determined. Secondly, this study solely examined the initial ROX index within a 24-hour timeframe, leaving the association between the fluctuating ROX index and outcomes uncertain. Finally, we fitted the segmented linear model based on whether ROX is less than 8.28, but this number is not very precise and requires more rigorous experimental design and more suitable statistical approaches to obtain a more accurate threshold.

## Conclusion

In summary, the findings of our study demonstrate that the ROX index serves as a valuable predictor of mortality risk in adult patients with AHRF, and that a lower ROX index is substantially associated with an increase in mortality.

## Data Availability

The datasets used and/or analyzed during the current study are available from the corresponding author on reasonable request.

## Abbreviations

MIMIC-IV: Medical Information Mart for Intensive Care IV
AHRF: Acute hypoxemic respiratory failure
HFNC: High flow nasal cannula
RR: Respiratory rate
HR: Hazard ratio
CI: Confidence intervals
BMI: Body Mass Index
SOFA: Sequential Organ Failure Assessment
SIRS: Systemic inflammatory response syndrome
SAPS II: Simplified acute physiological score II
MBP: Mean blood pressure
ALT: Alanine aminotransferase
AST: Aspartate aminotransferase
BUN: Blood urea nitrogen
INR: International normalized ratio
PT: Prothrombin time
PTT: Partial thromboplastin time
